# Microbiome analysis reveals the inducing effect of *Pseudomonas* on prostatic hyperplasia via activating NF-κB signalling

**DOI:** 10.1080/21505594.2024.2313410

**Published:** 2024-02-20

**Authors:** Jiaren Li, Youyou Li, Liang Zhou, Hongming Li, Tengfei Wan, Jin Tang, Lei Zhou, Hui Xie, Long Wang

**Affiliations:** a Department of Urology, The Third Xiangya Hospital, Central South University, Changsha, Hunan, China; b Movement System Injury and Repair Research Center, Xiangya Hospital, Central South University, Changsha, Hunan, China

**Keywords:** Benign prostatic hyperplasia, microbiome, *Pseudomonas*, lipopolysaccharide, NF-κB

## Abstract

Benign prostatic hyperplasia (BPH) is a prevalent disease among middle-aged and elderly males, but its pathogenesis remains unclear. Dysbiosis of the microbiome is increasingly recognized as a significant factor in various human diseases. Prostate tissue also contains a unique microbiome, and its dysbiosis has been proposed to contribute to prostate diseases. Here, we obtained prostate tissues and preoperative catheterized urine from 24 BPH individuals, and 8 normal prostate samples as controls, which followed strict aseptic measures. Using metagenomic next-generation sequencing (mNGS), we found the disparities in the microbiome composition between normal and BPH tissues, with *Pseudomonas* significantly enriched in BPH tissues, as confirmed by fluorescence in situ hybridization (FISH). Additionally, we showed that the prostate microbiome differed from the urine microbiome. In vitro experiments revealed that lipopolysaccharide (LPS) of *Pseudomonas* activated NF-κB signalling, leading to inflammation, proliferation, and EMT processes, while inhibiting apoptosis in prostatic cells. Overall, our research determines the presence of microbiome dysbiosis in BPH, and suggests that *Pseudomonas*, as the dominant microflora, may promote the progression of BPH through LPS activation of NF-κB signalling.

## Introduction

Benign prostatic hyperplasia (BPH) is a common condition among middle-aged and elderly males, which becomes the primary aetiology for lower urinary tract symptoms (LUTS) [1]. The prevalence of BPH rises substantially with age, which starts at age 40–45 years, escalating to 60% by age 60, and 80% by age 80 [2]. In the histopathological examination of BPH, the observed augmentation in cell quantities can be attributed to either the proliferation of prostatic cells or the hindered process of programmed cell death [3]. However, its specific mechanisms have not been elucidated.

The human microbiome comprises a diverse assemblage of microorganisms, encompassing bacteria, fungi, viruses, and parasites that maintain symbiotic relationships in the body, including the prostate [4]. However, the human microbiome may alter due to a variety of internal and external environmental factors, which causes microbiome dysbiosis and is closely associated with diseases [5]. Emerging evidences confirm this influence of the human microbiome on human health and development of diseases [6,7]. Previous research has demonstrated that the microbiome can affect prostate inflammation, which is associated with prostate diseases including BPH and prostate cancer (PC) [8,9]. A study showed that there were disparities in urine microbiome between BPH patients and normal subjects, and the ecological imbalance of urine microbiome may be connected with the occurrence of BPH [10]. Moreover, dysbiosis of the microbiome has already been found in PC tissues [11]. Another study documented that bacterial constituent lipopolysaccharide (LPS) can initiate the activation of NF‐κB in PC cells, promoting their survival and metastasis [12]. However, there remains a lack of comprehensive understanding regarding the involvement of the prostate microbiome in prostate diseases.

To ascertain the presence of microbiome dysbiosis in BPH tissues and explore the potential contribution of the local microbiome in influencing the BPH progression, metagenomic sequencing was performed on collected BPH and normal prostate tissues. We revealed that there was a higher relative abundance of *Pseudomonas* in BPH tissues, and LPS of *Pseudomonas* may exert a significant influence on the progression of BPH via activating NF-κB signalling.

## Materials and methods

### Human prostate tissue samples collection

The patients had no infection, antibiotics, or catheterization 3 months before the surgery. All samples were collected under strict aseptic conditions to ensure no interference from exogenous microorganisms. We obtained both catheterized urine and hyperplastic prostate tissue from patients (*n* = 24) who were admitted for transurethral prostate resection (TURP) surgery (samples exhibited BPH without tumour infiltration). Normal prostate samples (*n* = 8) were obtained from bladder cancer patients after radical cystectomy (age <60; total PSA <4 ng/ml; prostates were normal in size and shape, without tumour infiltration). To ensure that the samples were negative for cancer, the prostate pathology examination was conducted by two histopathologists operating independently (the cellular morphology and size were normal; immunohistochemistry showed that tumour markers P504s and PMSA were negative, while HCK and p63 were positive). Table 1 presents the background characteristics of the patients. All prostate samples were obtained from the Third Xiangya Hospital of Central South University with written informed consent, and the collection procedure was authorized by the Institutional Review Board of Third Xiangya Hospital of Central South University.

**Table 1.** Baseline characteristics of patients with or without BPH expressed as median with interquartile range (*n* = 32).

Groups	Age (years)	BMI (kg/m^2^)	Prostate volume (mL)	IPSS
Normal (*n* = 8)	51.0 (49.0–55.3)	23.1 (20.6–23.7)	24.8 (22.7–25.2)	1.5 (0.3–2.8)
BPH (*n* = 24)	71.0 (67.3–73.0)	23.2 (20.6–24.2)	63.9 (54.3–75.6)	23.0 (19.3–26.0)
Total (*n* = 32)	68.5 (60.0–72.0)	23.1 (20.6–24.1)	59.1 (31.4–71.4)	21.0 (7.0–25.8)

### DNA extraction, library preparation, and sequencing

We used a Mag-Bind Universal Pathogen 96 kit (Omega Bio-Tek, USA) to extract the total DNA. Qubit dsDNA assay kit (Yeasen, China) was used to determine DNA concentration and quality. The concentration of 300ng DNA was utilized for the preparation of the whole Metagenome library (Library Prep Kit for MGI high-throughput sequencing platform). The DNA of 100–200 base pairs in size were fragmented and end-repaired using restriction enzymes. Subsequently, the fragments were ligated with the adaptor and the resulting library was enriched through 12 cycles of PCR. Purification was performed using Agencourt AMPure XP beads (Beckman Coulter), and the quality of the purified samples was assessed using the Qsep100 fragment analyser. The sequencing was performed by the DNBSEQ-T7 sequencer (BGI, Shenzhen, China) and the target amount of sequence data was 10 G per sample. To assess and eliminate the extra contamination from laboratory settings, reagents, DNA extraction kits (kitome), and other sources, we adhered to rigorous aseptic practices and used DNA-free water as the negative parallel control. The DNA-free water was involved in the whole process, from DNA extraction to sequencing. The raw data of DNA-free water only consisted of 252 reads (Table S1). And following data quality control measures and the exclusion of human sequence data, no microbial reads were left, indicating that additional contamination had been well controlled.

### Sequencing data analysis

The KneadData software (version 0.10.0) was employed to perform quality control on the raw data and to remove host-related sequences [13]. The assessment of the overall quality of each sample was conducted by FastQC (version 0.11.7) with default settings. The Trimmomatic (version 0.36) was employed to eliminate low-quality reads and those with a length shorter than 50 base pairs (MINLEN:50). The Bowtie2 (version 2.3.4.1) (–very-sensitive-local) was used to identify and exclude the sequence data of human aligning to the human (GRCh38 and human contamination) reference databases. The human contaminants were based on the study “Human contamination in bacterial genomes has created thousands of spurious proteins” [14]. Approximately 99% of the human sequences were eliminated, leaving only 1% for the classification. The taxonomic classification of the remaining reads was then performed by Kraken2 (version 2.1.1) with default settings. We used the “kraken2-build” command to create the standard Kraken2 database, the data was from (https://ftp.ncbi.nlm.nih.gov/), which contained NCBI taxonomic information, as well as the complete genomes in RefSeq for the bacterial, archaeal, and viral domains, along with the human genome and a collection of known vectors (UniVec_Core). About 36% of the reads on average were classified as homo, and this portion of the data was excluded from subsequent analyses. And taxonomic abundance was then normalized by Bracken 2.6 based on Kraken2 output [15]. Microbial taxa with relative abundances below 0.01% were excluded.

### Fluorescent in situ hybridization (FISH)

FISH was implemented on prostatic tissue sections using *Pseudomonas* genus specific probes (5’-GATCCGGACTACG ATCGGTT-3’). The prostatic tissues were embedded in OCT (Optimal Cutting Temperature) compound and subsequently sectioned while frozen at a thickness of 10 μm. Prior to being fixed in a 4% paraformaldehyde solution, the sections were allowed to thaw at ambient temperature. The sections were rinsed with PBS, and then a probe volume of 2 μL was combined with 98 μL of FISH buffer. The resulting solution was applied to the section (50–100 μL), which subsequently positioned in a hybridization chamber (37 °C) for 24 h. The sections underwent two rounds of washing and were then incubated at 48 °C for 15 min. Subsequently, the sections were air-dried at ambient temperature. Prior to visualization, DAPI and Vectashield were applied onto the slides. Slides were scrutinized using a Zeiss fluorescence microscope (Jena, Germany) and the acquired images were processed using ImageJ. And the quantification of *Pseudomonas* probe reactivity was conducted by determining the percentage (%) of cells exhibiting perinuclear probe reactivity, with a minimum count of 200 cells.

### Haematoxylin-Eosin (H&E) staining and immunohistochemistry (IHC)

In the process of H&E staining, the sections underwent a dewaxing procedure, followed by a 5-minute staining in haematoxylin. Subsequently, the sections were subjected to three washes with water. Then, a 30-second staining in eosin was performed, followed by dehydration and mounting using conventional techniques. For IHC, the sections underwent de-paraffinization and hydration, after which antigen retrieval was conducted using a pH = 6.0 citrate buffer by incubating the sections in a steamer for 30 minutes. Then, the sections were subjected to three washes with PBS, and a 3% H_2_O_2_ solution was used to quench endogenous peroxidase. To prevent non-specific binding, the slides were incubated for 30 minutes with bovine serum, and then subjected to an overnight incubation at 4°C with the primary antibody (anti-IL-6, dilution 1:200, #66146–1, Proteintech; anti-COX-2, dilution 1:200, #66351–1, Proteintech; anti-p65, dilution 1:400, #8242, Cell Signaling Technology). Then, the slides were subjected to incubation with a biotinylated secondary antibody specific to rabbit/mouse immunoglobulin G (IgG) for 1 hour. The resulting signal was visualized using the DAB Substrate Kit (Yeasen). Slides were then counterstained using haematoxylin, air-dried, and permanently mounted with neutral resin. An Olympus C×31microscope (Tokyo, Japan) was used to observe stained slides and capture images.

### Analysis of dataset GSE179655

The dataset GSE179655 was obtained from the Gene Expression Omnibus (GEO, https://www.ncbi.nlm.nih.gov/geo/) database of National Center for Biotechnology Information (NCBI). The experimental groups were male C57 mice with urethral instillation of uropathogenic *E. coli* (UTI89) (samples GSM5425746, GSM5425747, GSM5425748) and control mice were instilled with saline (samples GSM5425743, GSM5425744, GSM5425745). The differentially expressed genes (DEGs) in the experimental and control groups were screened using the EdgeR package (version 3.16.5), with |log2FC| >1 and *p* < 0.05 considered significant [16]. Subsequently, the volcano map was visualized by the ggplot2 (version 3.3.6) in the R software (version 4.2.0), which showed the differential expression data.

### Cell culture

The BPH-1 cell line, derived from human benign prostatic enlargement epithelia, was obtained from Merck (SCC256) and cultured in RPMI-1640 medium (Merck) supplemented with 10% foetal bovine serum (FBS) (Merck). All cells were cultured in a humidified environment consisting of 95% air and 5% CO_2_ at 37°C.

### Western blot analysis

Protein extraction and western blot assays were conducted according to the methods previously outlined [3]. The p65 (#8242), phospho-p65 (Ser536, #3033), PCNA (#2586), Bcl-2 (#15071), BAX (#41162), E-cadherin (#3195), N-cadherin (#13116), and vimentin (#5741) primary antibodies were acquired from Cell Signaling Technology (MA, USA), while β-actin (HC201–01) was obtained from TransGen Biotech (Beijing, China). All antibodies are diluted 1:1000.

### RNA extraction and quantitative real-time PCR (qRT-PCR)

TRIzol Reagent (Takara) was used to isolate the total RNA. The quantification of RNA concentration was accomplished through the assessment of optical density at 260 nm and 280 nm wavelengths. 1 μg of the total RNA was employed for the production of cDNA by the First Strand cDNA Synthesis kit (Bimake). Then, we performed qPCR on an ABI PRISM® 7900HT System (Applied Biosystems). The relative gene expression was assessed using the relative standard curve method (2^−ΔΔCT^), with Gapdh serving as the reference gene. The primer sequences were documented in Table S2. Each sample was subjected to independent analysis, which was performed three times.

### Cell counting kit-8 (CCK-8) assay

The examination of cell viability was conducted by the CCK-8 assay (MedChemExpress). The BPH-1 (about 5000 cells/well) were cultured in 96-well plates and intervened accordingly. Then, CCK-8 solution (10 μl/100 μl) was introduced into each culture well, and BPH-1 cells were cultured at 37°C for 2 h. A microplate reader (Varioskan LUX; Thermo Fisher Scientific) was employed to measure the absorbance at 450 nm.

### EdU assay

A yefluor 647 EdU imaging kit (Yeasen) was employed to assess the proliferative capacity of the cells. A Zeiss fluorescence microscope (Jena, Germany) was used to obtain the images.

### Flow cytometry analysis

The apoptosis determination was conducted using an Annexin V-Alexa Fluor647/PI apoptosis detection kit (Yeasen). The collected cells were suspended in 500 μl binding buffer and subsequently cultured with 5 μl Annexin V-Alexa Fluor647 and 10 μl PI for 15 min in a light-restricted environment. Subsequently, the cells underwent assessment through the Flow cytometry (NovoCyte, Agilent, USA).

### Statistical analysis

The data values were shown as mean ± standard deviation (SD). Student’s *t*-test, one-way analysis of variance (ANOVA) and Wilcoxon rank sum test were employed for statistical analysis. Difference was deemed statistically significant at *p* < 0.05.

## Results

### Comparison of biodiversity indices between prostatic hyperplasia and normal prostate tissues

**Figure 1.**
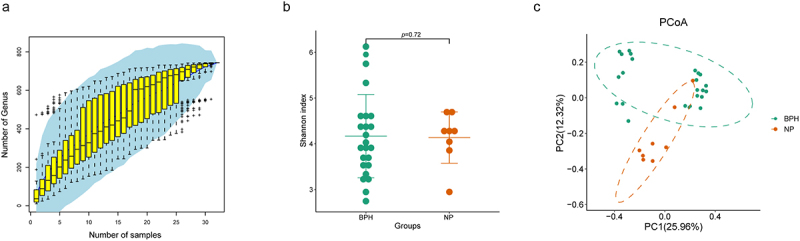
Biodiversity indices across distinct groups of samples. (a) the species accumulation curve of the sample is used to judge whether the samples size is sufficient and estimate the species richness. (b) Shannon index reveals the alpha-diversity in prostate microbiome of BPH and normal tissues (*p* = 0.72, Wilcoxon rank sum test). (c) PCoA based on the Bray-Curtis distance shows the beta-diversity in prostate microbiome of BPH and normal tissues.

We established a study cohort included BPH tissue (*n* = 24) and normal prostate tissue (*n* = 8) as controls. The stability of the species accumulation curve observed in the samples provides evidence that all samples were subjected to sequencing procedures that ensured high sequence depth and richness (Figure 1a). Alpha-diversity reflects the samples’ internal diversity and species richness. In our study, Shannon index was used to estimate the richness and evenness of prostate microbiome. Our findings indicate that there is no disparity in the Shannon index between the two groups (*p* = 0.72, Wilcoxon rank sum test, Figure 1b). Additionally, we inquired about potential disparities in microbial communities by employing beta-diversity, a metric that elucidates the divergences in microbial compositions across various groups. Principal Co-ordinates Analysis (PCoA) based on the Bray-Curtis distance shows the disparity between BPH and normal prostate tissues in beta-diversity, with prostate microbiome communities of the two groups forming separate clusters (Figure 1c).

### Microbiome composition is altered between prostatic hyperplasia and normal prostate tissues

**Figure 2.**
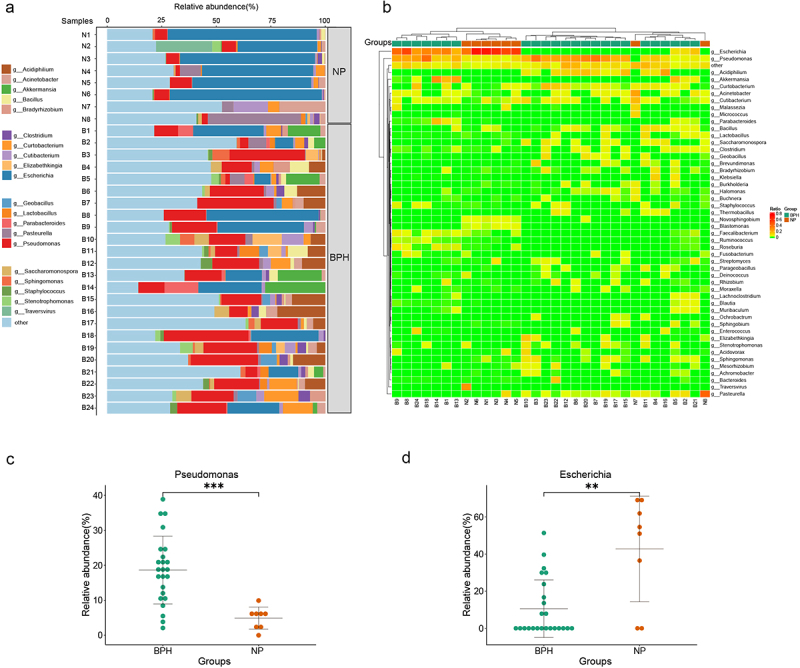
Bacterial composition of prostate tissue microbiome identified by metagenomic sequencing. (a) the bar plots are used to show the relative abundances of bacterial genera across various prostate tissue samples. (b) Heatmap of genus-level abundance in sequencing data, based on the Euclidean distance and complete-linkage hierarchical cluster. (c) dot plot showing the relative abundance (%) of *Pseudomonas*. (d) dot plot showing the relative abundance (%) of *Escherichia*. Wilcoxon rank sum test; **p < 0.01; ***p < 0.001.

To examine the factors contributing to variations in microbiome diversity and gain a deeper understanding of the alterations in microbiome structure between BPH and normal prostate tissues, we employed bar plots to illustrate the composition of dominant bacteria in both groups and to examine modifications in microflora at the genus level (Figure 2a). Moreover, the heatmap based on the Euclidean distance and complete-linkage hierarchical cluster shows the species composition and variations among the samples (Figure 2b). The dominant genera observed in normal prostate tissues are *Escherichia* (*p* < 0.01, Wilcoxon rank sum test) corresponding to the significantly increased abundance of *Pseudomonas* (*p* < 0.001, Wilcoxon rank sum test) in BPH samples (Figure 2c, d). As the bacteria genus with the highest abundance in BPH tissues, we speculated that *Pseudomonas* may be a potential influencing factor in the pathogenesis of BPH. Furthermore, we conducted Spearman correlation analysis to assess the relationship between *Pseudomonas* abundance in prostate tissue of BPH patients and their age, BMI, International Prostate Symptom Score (IPSS), and prostate volume (supplementary figure S1). The results reveal that the abundance of *Pseudomonas* does not exhibit any significant correlation with age (*R* = −0.052, *p* = 0.808), BMI (*R* = −0.150, *p* = 0.485) or IPSS (*R* = 0.289, *p* = 0.171). While there is a positive correlation trend with prostate volume (*R* = 0.387, *p* = 0.062), this correlation does not reach statistical significance.

### The prostate microbiome differs from urine microbiome

**Figure 3.**
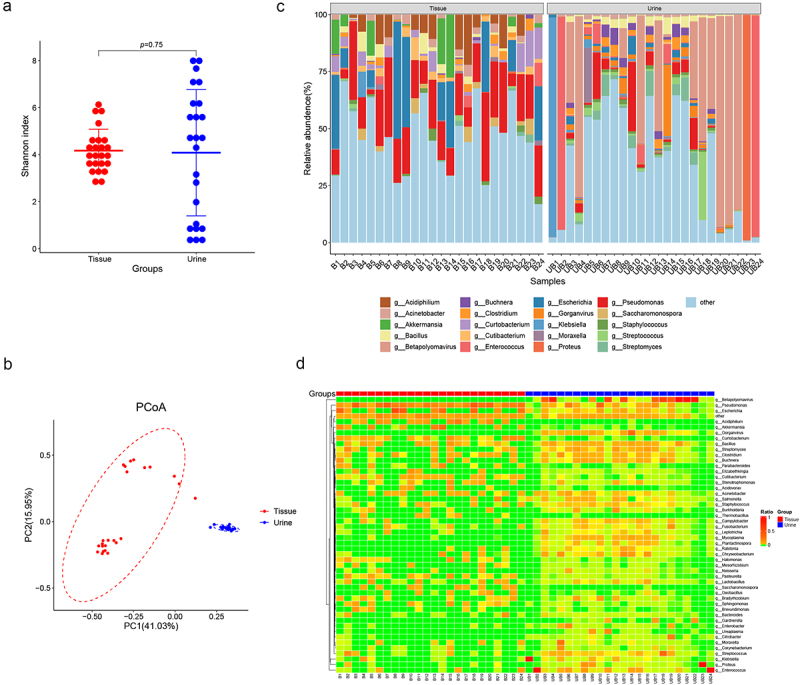
Comparisons of microbiome in prostate tissue and catheterized urine. Shannon index (a) shows alpha-diversity (*p* = 0.75; Wilcoxon rank sum test) and PCoA based on the Bray-Curtis distance (b) shows beta-diversity. (c) the relative abundances of bacterial genera in various samples shown in bar plots. (d) Heatmap of genus-level abundance in prostate tissue and catheterized urine, based on the Euclidean distance and complete-linkage hierarchical cluster.

Additionally, we conducted a comparative analysis between the microbiome present in BPH tissue and that in catheterized urine. The result suggests that there is no difference in the Shannon index between the tissue and urine microbiome (*p* = 0.75, Wilcoxon rank sum test, Figure 3a). Moreover, PCoA reveals a distinction in the microbiome composition between prostate tissue and urine (Figure 3b). The abundant bacteria genera in BPH tissues, including *Pseudomonas*, *Acidiphilium* and *Akkermansia*, were not similarly dominated in urine samples. In contrast, urine samples showed higher levels of *Betapolyomavirus*, *Gorganvirus*, and *Bacillus*, which were rare in tissues (Figure 3c, d).

### *Pseudomonas* is present in prostate tissue and may cause prostate inflammation

**Figure 4.**
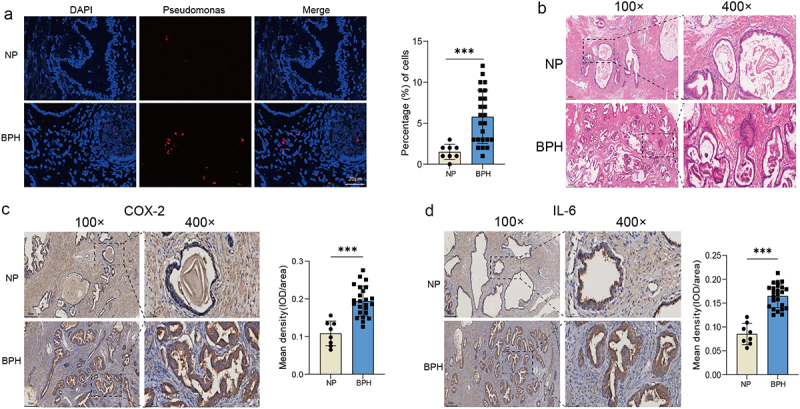
The presence of *Pseudomonas* and degree of inflammation in prostate tissues. (a) Representative FISH images of *Pseudomonas* in prostate tissues, and quantification of *Pseudomonas* probe reactivity comparing the percentage (%) of cells with perinuclear probe reactivity in BPH and normal prostate tissues. (b) Representative H&E staining images of prostate tissues. Representative images of IHC for COX-2 (c) and IL-6 (d) in prostate sections from BPH and normal groups. Quantification bar graph showing the extent of COX-2 and IL-6 staining in the form of mean density calculated by IOD/area. Data are shown as mean ± SD, Student’s *t*-test; ***p < 0.001.

The sequencing data revealed a dominance of *Pseudomonas* in BPH tissue, prompting our subsequent objective to validate this finding through FISH. Using fluorescent probe specific to *Pseudomonas*, we found that there was indeed a certain amount *Pseudomonas* in BPH tissue, whereas a sparse signal in normal prostate. And the discrepancy exhibited statistical significance (*p* < 0.001, Student’s *t*-test, Figure 4a). Presence of viable bacteria and bacterial components, such as LPS, has been shown to induce inflammation, which is confirmed the association with the pathogenesis and progression of BPH [17]. Therefore, we stained the samples to check the levels of inflammation. H&E staining showed the normal prostate tissue and BPH tissue with increased glandular epithelial thickness and stromal components (Figure 4b). Moreover, we found through IHC staining that the expressions of two inflammatory factors, COX-2 and IL-6, were visibly increased in BPH tissue (*p* < 0.001, Student’s *t*-test, Figure 4c, d).

### NF-κB may be a key factor involved in microbiome dysbiosis-associated BPH

**Figure 5.**
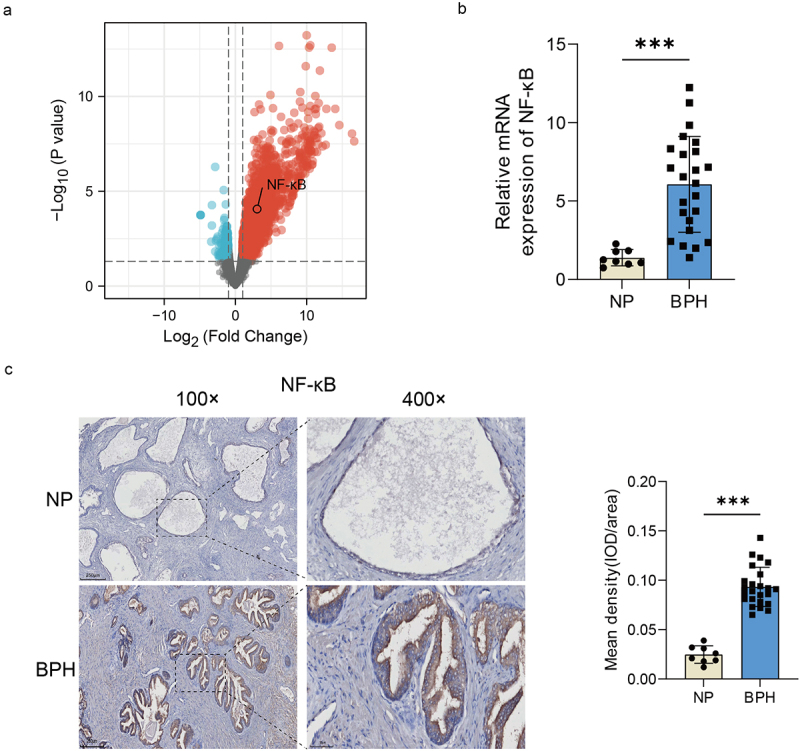
The increase of NF-κB expression in BPH tissues. (a) Volcano map of DEGs after urethral bacterial instillation based on GSE179655. blue dots: down-regulated genes; red dots: up-regulated genes; gray dots: no significant difference. (b) transcription levels of NF-κB by qRT-PCR in BPH and normal tissues. (c) Representative images of IHC for NF-κB in prostate sections from BPH and normal groups. Quantification bar graph showing the extent of NF-κB staining in the form of mean density calculated by IOD/area. Data are shown as mean ± SD, Student’s *t*-test; ***p < 0.001.

Gram-negative bacteria and the LPS produced by them, are reported to be the important inflammatory inducers that can cause a variety of diseases via activating the TLR4-NF-κB pathway [18]. And the involvement of NF-κB in the pathogenesis of BPH has been documented [19]. Therefore, we hypothesized that the activation of NF-κB by bacteria and the LPS may play an important role in the development of BPH. Popovics et al. used the uropathogenic *E. coli* (UTI89), a serotype that can cause urinary tract infections, to establish the mouse model of prostatic inflammation, and performed RNA sequencing of the obtained prostates (data was uploaded to the dataset GSE179655) [16]. We analysed and visualized the DEGs between the experimental and control groups of the dataset GSE179655, and generated a volcano map to depict the results, which revealed a notable increase in NF-κB expression within the prostate following bacterial induction (Figure 5a). We further verified the collected samples by qRT-PCR (*p* < 0.001, Student’s *t*-test, Figure 5b) and IHC (*p* < 0.001, Student’s *t*-test, Figure 5c), and found that NF-κB expression was indeed elevated in BPH group. These results indicated that NF-κB signalling could potentially be involved in prostatic inflammation and BPH induced by bacteria or their LPS.

### In vitro experiments show that LPS of *Pseudomonas* can influence the BPH-1 cell phenotypes via activating NF-κB signalling

**Figure 6.**
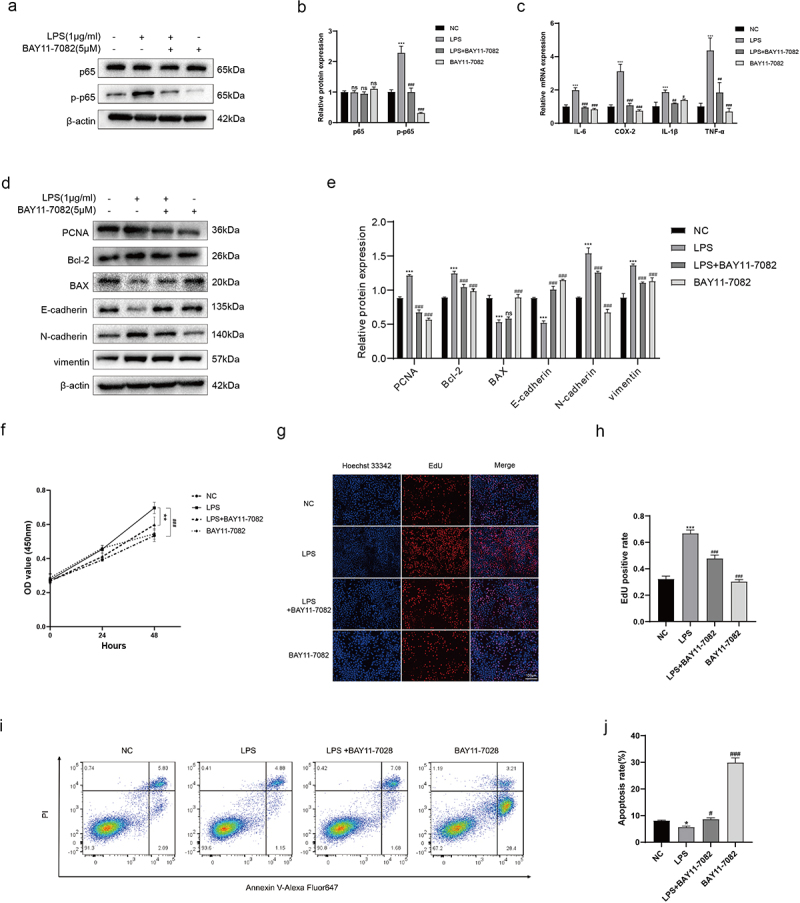
LPS of *Pseudomonas* affects the cell phenotypes via activating NF-κB. (a, b) BPH-1 cells were cultured by LPS (1 μg/ml) and/or BAY-11-7082 (5 μM) for 48 h, comparing with control cells; western blot and relative densitometric quantification for total and phosphorylated p65. (c) transcription levels of inflammatory cytokines by qRT-PCR at the above conditions. (d, e) western blot and relative densitometric quantification for proliferative protein (PCNA), apoptotic proteins (bcl-2, BAX) and EMT proteins (E-cadherin, N-cadherin, and vimentin). CCK-8 assay (f) and EdU assay (g, h) were used to analyze the cell proliferation of BPH-1 cells. (i) flow cytometry analysis was used to detect apoptotic cells stained with annexin V and PI, and the resulting apoptotic rates were subjected to statistical analysis (j). Data are shown as mean ± SD, one-way ANOVA; *p < 0.05, **p < 0.01, and ***p < 0.001: compared to the NC group; ^#^*p* < 0.05, ^##^*p* < 0.01, and ^###^*p* < 0.001: compared to the LPS group.

As mentioned above, we designed in vitro experiments to provide a more comprehensive understanding of NF-κB in *Pseudomonas* induced BPH. Recombinant LPS derived from *Pseudomonas* was employed for cell culturing (BPH-1), and the expression level of corresponding proteins were detected by Western blot. Our results indicated that the p-p65 (the primary transcription subunit of NF-κB) expression was significantly increased in LPS-interfered cells, which could be reversed by BAY-11-7082 (an inhibitor for p65 phosphorylation) (Figure 6a, b). Subsequently, we quantified the transcription levels of inflammatory factors using qRT-PCR, and revealed that LPS could increase the expression of common inflammatory factors (IL-6, COX-2, IL-1β, TNF-α), which could also be inhibited by BAY-11-7082 (Figure 6c). In addition, NF-κB can directly participate in the process of cell proliferation, apoptosis and EMT, which are closely related to the BPH [20,21]. Therefore, we then examined the expression of proliferative protein (PCNA), apoptotic proteins (Bcl-2, BAX) and EMT proteins (E-cadherin, N-cadherin, and vimentin), revealing that LPS could promote proliferation and EMT of BPH-1 cells and inhibit their apoptosis (Figure 6d, e). Moreover, we determined that LPS can accelerate cell proliferation using CCK-8 assay (Figure 6f) and EdU assay (Figure 6g, h). As for cell apoptosis, flow cytometry analysis was used to verify that LPS can indeed reduce apoptosis (Figure 6i, j). All these trends could be rescued by BAY-11-7082.

## Discussion

The emergence of high-throughput sequencing techniques, exemplified by mNGS, has facilitated the identification of the microbiome’s existence in various organs and tissues within the human body [7,22]. In 2007, the Human Microbiome Project (HMP) was launched in an attempt to investigate the human microbiome for gaining a deeper comprehension of its significance in relation to human health and diseases [23]. Various factors like age, exercise, diet, smoking, medications may contribute to the microbiome composition of our body, which lead to organ lesions and diseases [5,6,24]. Conversely, changes in the abundance and composition of microorganisms at specific sites under diseased conditions can also lead to further disease progression, and can serve as potential biomarkers of disease [25].

BPH is a complicated and multifactorial disease involving genetic, androgenic, and inflammatory factors [26]. As an important pro-inflammatory factors, microbiome has been substantiated to contribute to the advancement of BPH. Gut microbiome, the largest source of human microorganisms, was initially found to affect BPH through the “gut-prostate axis” [27,28]. Takezawa et al. demonstrated that the prostate enlargement (PE) was linked with *Firmicutes/Bacteroidetes* (F/B) ratio in the human gut microbiome, which was notably higher in the PE group compared to the non-PE group [29]. The F/B ratio is a considerable indicator of gut microbiome conditions, since it is associated with numerous diseases. Gu et al. found that the F/B ratio within the gut microbiome of mice subjected to a high-fat diet was significantly higher in a BPH mouse model induced by testosterone propionate [30]. However, our findings suggest that there was no statistical difference in the F/B ratio on prostate tissues, and thus it cannot be used to reflect the disease status of the prostate (Figure S2).

While conclusive evidence is lacking, studies have suggested a potential association between certain bacterial species found in urine and prostate diseases. A study included 77 individuals diagnosed with BPH and 30 control participants demonstrated there was a significant disparity in urine microbiome between the two groups [10]. Specifically, the bacterial genera *Staphylococcus*, *Bacillus*, *Finegoldia*, *Haemophilus*, and *Listeria* in urine samples were associated with high IPSS scores. In our study, *Staphylococcus* and *Bacillus* were also observed in the urine of patients with BPH, providing further support for the above findings.

Compared to the microbiome of the gut and urine, there has been less research on the microbiome on human tissues. For one reason, there are strict restrictions and ethical requirements on the acquisition of tissue specimens. Meanwhile, the microbiome abundance in tissues is relatively rare, and uninfected tissues could not be identified microbially until high-throughput sequencing techniques were developed. However, as the microbiome that comes into direct contact with organs, it also plays a crucial role in the course of disease.

We found that the genus *Pseudomonas* was dominant in BPH tissues, which is a multi-drug resistance (MDR) opportunistic pathogen, and can cause infection in patients with compromised immune systems [18]. In the genitourinary system, there is a direct correlation between *Pseudomonas* and total motile sperm count [31]. *Pseudomonas* is known to possess a high concentration of regulatory genes, which may enable it to acclimate to environmental alterations and withstand antimicrobial agents [32]. Perhaps these adaptive traits permit the persistence of certain *Pseudomonas* bacteria within the safeguarded prostate tissue.

LPS, a major virulence factor secreted by *Pseudomonas*, can be identified by diverse host receptors to trigger inflammatory and immune responses, and is the leading cause of chronic tissue damage [33]. Additionally, LPS has been shown to accelerate chondrocyte migration and proliferation, as well as inhibit chondrocyte apoptosis [34]. LPS can also regulate the EMT-mediated airway remodelling to induce respiratory tract infections [35]. The transcription factor NF-κB holds significant importance in cell inflammation, proliferation and EMT [20,21]. Previous studies documented that the NF-κB activation promoted the persistent transcription of genes associated with proliferation by sustaining the functionality of androgen receptors, thereby exerting a pivotal influence on BPH [19,36]. Furthermore, LPS possesses the capacity to stimulate the generation of NF-κB in a TLR4-dependent manner [18]. In our research, LPS activated the phosphorylation of NF-κB, thereby promoting prostatic cells inflammation, proliferation, and EMT processes. Collectively, these findings underscore the significance of NF-κB as a crucial modulator of LPS- induced promotion of BPH.

Notwithstanding the merits of our study, it is imperative to acknowledge the presence of several limitations. None of the individuals had received other antibiotics 3 months prior to surgery, but to mitigate the risk of postsurgical infection, all the patients were subjected to antibiotic treatment 30 minutes before the commencement of surgery, which might have some influence on the results. Additionally, our study included a relatively small number of participants. Further research involving a larger sample size is required to confirm our findings. Furthermore, we only focused on bacteria and ignored analysis on viruses and fungi, which also hold significance in shaping the profile of the prostate microbiome and should be taken into account in forthcoming research endeavours.

Overall, this study investigated the role of microbiome dysbiosis in BPH. Using metagenomic sequencing technique, we identified the differences in microbiome composition between normal and BPH tissues, with *Pseudomonas* being particularly enriched in BPH tissues. Furthermore, *Pseudomonas*-derived LPS activated NF-κB signalling, leading to inflammation, proliferation and EMT processes, while inhibiting apoptosis in prostatic cells. This research sheds light on the potential mechanisms underlying BPH progression and highlights the role of *Pseudomonas* and its LPS-induced pathways in driving the disease.

## Supplementary Material

Table S1.xlsx

Table S2.docx

Fig.S1.docx

FigS2.tif

## Data Availability

The sequencing data are available and have been uploaded to the National Center for Biotechnology Information (NCBI) under the BioProject ID PRJNA1061468.
