# Supplementary material for: Microbiome analysis reveals the inducing effect of Pseudomonas on prostatic hyperplasia via activating NF-κB signalling
Source: Virulence. 2024 Feb 20;15(1):2313410. doi: 10.1080/21505594.2024.2313410 (PMC10880505; doi:10.1080/21505594.2024.2313410)
Supplement: Table S2.docx [file KVIR_A_2313410_SM4878.docx]

**Table S2. The primer sequence for the study**

|  | **Forward (5’-3’)** | **Reverse (5’-3’)** |
| --- | --- | --- |
| RT-PCR  IL-6 | ACTCACCTCTTCAGAACGAATTG | CCATCTTTGGAAGGTTCAGGTTG |
| RT-PCR  COX-2 | CTGGCGCTCAGCCATACAG | CGCACTTATACTGGTCAAATCCC |
| RT-PCR  IL-1β | AGCTACGAATCTCCGACCAC | CGTTATCCCATGTGTCGAAGAA |
| RT-PCR  TNF-α | GAGGCCAAGCCCTGGTATG | CGGGCCGATTGATCTCAGC |
| RT-PCR  GAPDH | GCCTTCCGTGTTCCTACCCC | CGCCTGCTTCACCACCTTCT |
