# Supplementary material for: Microbiome analysis reveals the inducing effect of Pseudomonas on prostatic hyperplasia via activating NF-κB signalling
Source: Virulence. 2024 Feb 20;15(1):2313410. doi: 10.1080/21505594.2024.2313410 (PMC10880505; doi:10.1080/21505594.2024.2313410)
Supplement: Fig.S1.docx [file KVIR_A_2313410_SM4877.docx]

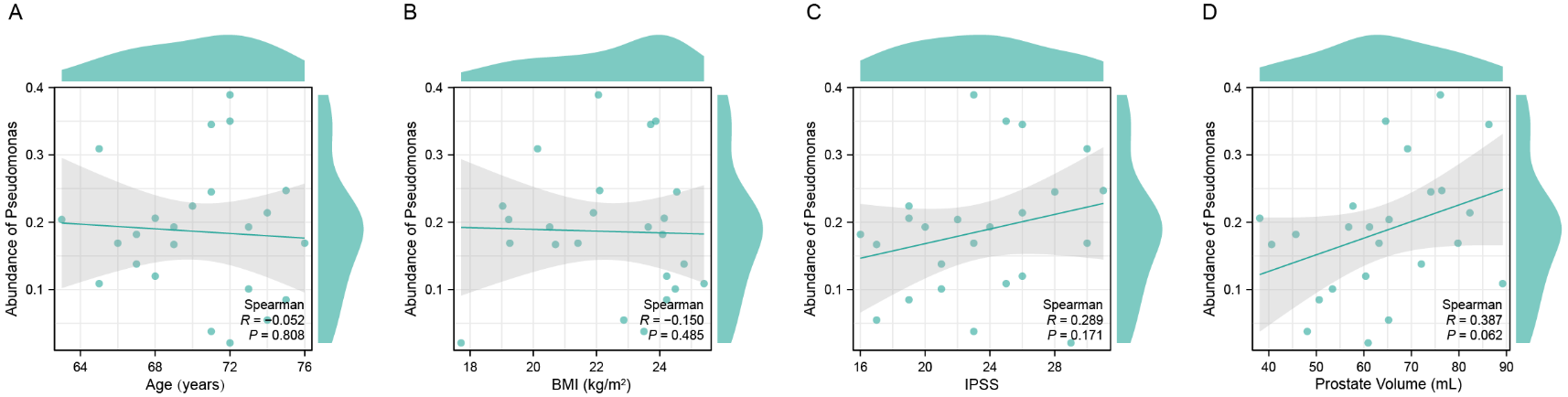


**Fig.S1** Abundance of *Pseudomonas* and clinical correlation. (**A**) *Pseudomonas* abundance did not exhibit significant correlation with age (R=-0.052, *p*=0.808). (**B**) *Pseudomonas* abundance did not exhibit significant correlation with BMI (R=-0.150, *p*=0.485). (**C**) *Pseudomonas* abundance did not exhibit significant correlation with IPSS (R=0.289, *p*=0.171). (**D**) *Pseudomonas* abundance exhibit a positive correlation trend with prostate volume, but this correlation did not reach statistical significance (R=0.387, *p*=0.062).
