# Supplementary figures and images for: Microbiome analysis reveals the inducing effect of Pseudomonas on prostatic hyperplasia via activating NF-κB signalling
Source: Virulence. 2024 Feb 20;15(1):2313410. doi: 10.1080/21505594.2024.2313410 (PMC10880505; doi:10.1080/21505594.2024.2313410)

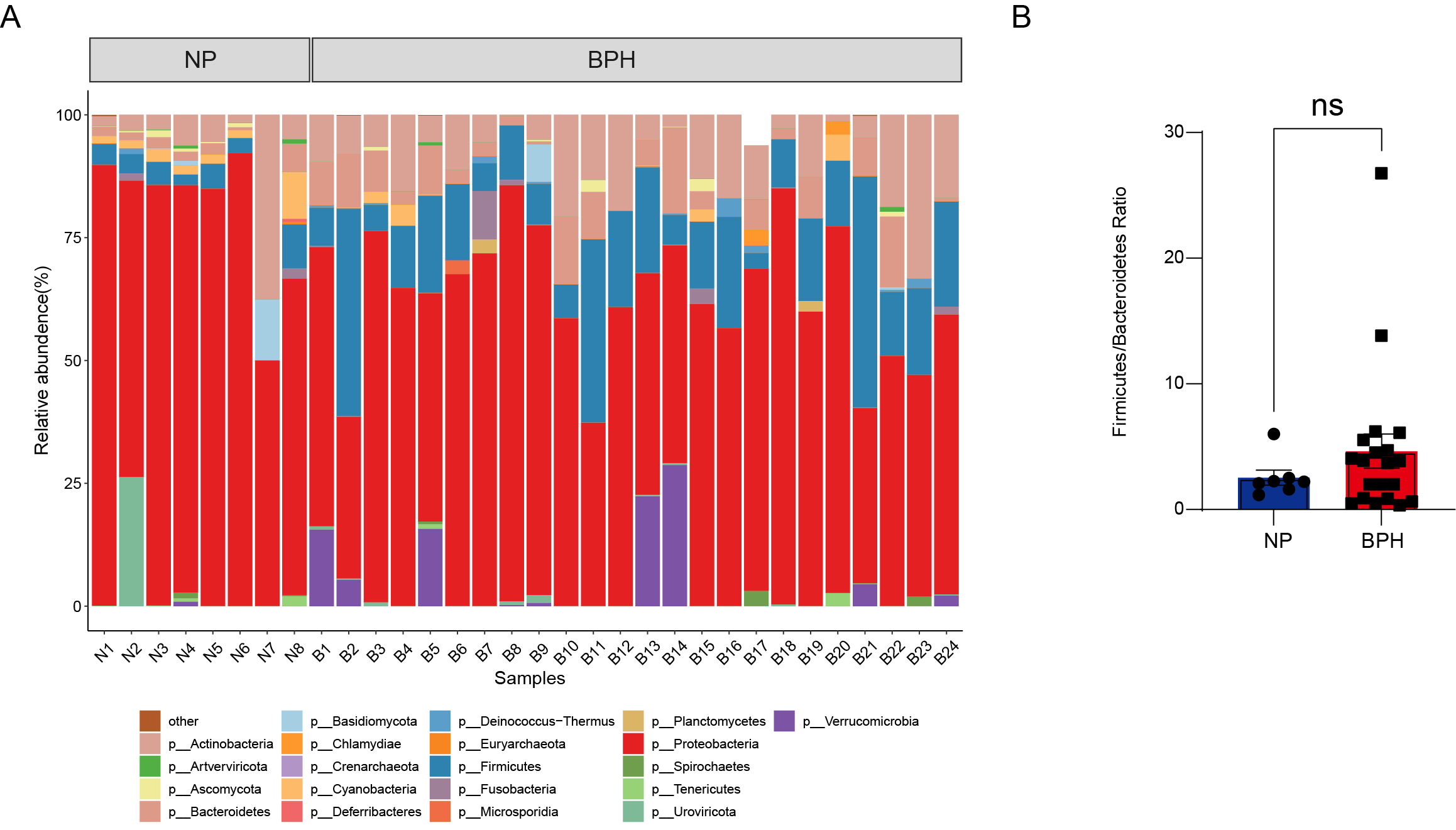

Supplement: FigS2.tif [file KVIR_A_2313410_SM4876.tif]
